# Tumor-associated macrophages (TAMs) depend on MMP1 for their cancer-promoting role

**DOI:** 10.1038/s41420-021-00730-7

**Published:** 2021-11-09

**Authors:** Junhui Yu, Zhengshui Xu, Jing Guo, Kui Yang, Jianbao Zheng, Xuejun Sun

**Affiliations:** Department of General Surgery, First Affiliated Hospital of Xi’an Jiaotong University, Xi’an, 710061 Province PR China

**Keywords:** Cancer microenvironment, Cell growth

## Abstract

The complex interaction between tumor-associated macrophages (TAMs) and tumor cells through several soluble factors and signaling is essential for colorectal cancer (CRC) progression. However, the molecular mechanism involved remains elusive. In this study, we demonstrated that MMP1 derived from TAMs markedly facilitated colon cancer cell proliferation via accelerating cell cycle transition from G0/G1 to S and G2/M phase. Moreover, exogenous MMP1 activated cdc25a/CDK4-cyclin D1 and p21/cdc2-cyclin B1 complexes through altering c-Myc and ETV4. Mechanistic studies indicated that inhibition of PAR1 or blockage of MAPK/Erk signaling eliminated the proliferation induced by exogenous MMP1 in vitro and in vivo. In addition, ETV4 could bind to the promoter of MMP1 and activate MMP1 transcription, which confirmed the MMP1/ETV4/MMP1 positive feedback. Altogether, our study identified a cytokine paracrine manner between colon cancer cells and TAMs. MMP1/PAR1/Erk1/2/ETV4 positive feedback loop may represent to be a therapeutic target and prognostic marker in CRC.

## Introduction

Colorectal cancer (CRC) ranks the second leading cause of cancer-related death [1]. Globally, ~1,800,000 new cases are diagnosed as CRC every year. Although great progress has been achieved in early detection and multimodality treatment of CRC [2, 3], most advanced CRC patients have a poor prognosis. Distant metastasis and relapse are the main cause of death for CRC patients [4, 5]. Emerging evidence confirms that the tumor microenvironment (TME), characterized by the interaction between tumor cells and the host, exerts a pivotal influence on tumor development and progression [6]. Therapeutic strategies targeting TME might complement traditional chemotherapy and enhance anti-tumor effect [7].

Tumor-associated macrophages (TAMs) are the most abundant component of the immune infiltration of TME [8]. The infiltration of TAMs is strongly associated with high vascular grade, reduced overall survival, and decreased relapse-free survival, and can serve as an independent prognostic indicator of cancers [9, 10]. By releasing various types of cytokines including growth factors, chemokines, and inflammatory factors, TAMs can endow tumor cell accelerative proliferative ability, strengthen chemotherapeutic resistance, increase motility and invasiveness and enhance immune escape capabilities [11, 12]. Matrix metalloproteinases (MMPs) secreted by TAMs, especially MMP2 and MMP9, are responsible for collagen degradation and tumor metastasis [13, 14]. MMP1 has been identified as an agonist of protease-activated receptor-1 (PAR1) [15, 16]. The co-expression of MMP1 and PAR1 is strongly related to tumor stage, lymphatic metastasis and tumor recurrence in human cancer [17, 18]. Activation of PAR1 triggers oncogenic transformation and accelerates tumor progression [19].

In the present study, we demonstrated that MMP1 derived from TAMs facilitates HT-29 and Caco-2 cell proliferation via accelerating cell cycle transition from G0/G1 to S and G2/M phase. Treatment with PAR1 inhibitor or knockdown of PAR1 eliminates the proliferation induced by exogenous MMP1. Mechanistic studies indicated that MMP1/PAR1 axis exerts its tumor promotion by activating MAPK/Erk pathway. In addition, ETV4 could bind to the promoter of MMP1 and activate MMP1 transcription, which confirmed the MMP1/ETV4/MMP1 positive feedback. In summary, our study identified a cytokine paracrine manner between colon cancer cells and TAMs. MMP1/PAR1/Erk1/2 pathway may represent to be a therapeutic target and prognostic marker in CRC.

## Materials and methods

### Cell cultures

All cell lines in the present study were procured from the Shanghai Institute of Cell Biology, Chinese Academy of Sciences (Shanghai, China). Cells are regularly checked for mycoplasma contamination before experiments. Cells were maintained in DMEM (HyClone, Logan, UT, USA) with 10% fetal bovine serum (HyClone, Logan, UT, USA). Incubator with 5% CO_2_-humidified at 37 °C was supplied for cell propagation.

### Activated macrophage induction and co-culture

U937 cells were induced into activated macrophages with PMA (10 ng/mL) and IL-4 (10 ng/mL) treatment [20]. Activated macrophages were seeded in the upper chamber with 0.4 μm pore (Corning Life Sciences, Lowell, MA, USA) and were co-cultured with HT-29 and Caco-2 cells in six-well plates at 37 °C and 5% CO_2_.

### TAMs isolation

For purpose of TAMs isolation, CRC tissues from patients underwent surgery were sliced up. The tissues were incubated with DMEM contained with ACCUMAX (Sigma‑Aldrich, Darmstadt, Germany) in a 5% CO_2_-humidified incubator at 37 °C for 2 h, subsequently percolated with 40 μm strainer (Thermo Fisher Scientific, Inc., Waltham, MA, USA). The cells were stained by primary antibody against CD68 (Abcam, Cambridge, MA, USA) to isolate and analysis TAMs. The isolated TAMs were seeded into a six-well plate for follow-up research. TAMs were cultured not exceeding 7 days.

### Conditioned media collection

The conditioned media (CM) derived from U937 or TAMs was filtered using 0.22 μm filter and was stored at −80 °C. HT-29 and Caco-2 cells was cultured with 0, 25, 50, and 75% of U937-CM or TAMs-CM for follow-up experiments.

### ELISA assay

MMP1 and MMP9 concentrations in CM derived from U937 or TAMs were measured using the QuantiCyto® Human MMP1 ELISA kit and QuantiCyto® Human MMP9 ELISA kit (#EHC134, EHC115, Neobioscience, Shenzheng, China) by following the manufacturer’s instructions.

### Vector construction and transfection

For MMP1 knockdown or overexpression, lentiviral vectors were synthesized by GeneChem Co., Ltd. (Shanghai, China). Lentiviral infection was processed following the manufacturer’s protocol.

The siRNA-PAR1 was purchased from Genepharma Co., Ltd. (Shanghai, China). SiRNA transfection was conducted using on Lipofectamine^TM^ 2000 (Invitrogen, Carlsbad, CA, USA), following the operation manual.

### Cell growth, cell viability, and cell cycle assays

Cells were seeded into 35-mm culture dishes, and cell numbers were calculated with a haemocytometer every other day to evaluate cell growth. To assess cell viability, cells at an initial quantity of 3000 cells/well were cultured in 96-well plates for 4 days, and CCK-8 assay (Dojindo, Tokyo, Japan) was performed every other day. For cell cycle analysis, 1 × 10^5^ cells were cultured in each well of six‑well plates firstly. Cell pellets were centrifuged and then fixed with 75% freezing ethanol overnight. After digested with RNase A and stained with PI for half an hour the following day respectively, cells were analyzed by flow cytometry (BD, Franklin Lakes, NJ, USA).

### Nude mouse xenograft assay

All animal experiments were approved by the Committee on the Ethics of Laboratory Animal Center of Xi’an Jiaotong University. The BALB/c-nude mice (5-week-old, female) were injected with 5 × 10^6^ cells subcutaneously. Nude mice were randomly divided into five groups (*n* = 5): (i) HT-29 + U937, (ii) HT-29 + TAMs, (iii) HT-29 + TAMs-shMMP1, (iv) HT-29 + TAMs + MK-5348, and (v) HT-29 + TAMs + SCH772984. MK-5348 and SCH772984 were administrated into nude mice via intraperitoneal injection every three days. Every 3 days, tumor volumes were calculated as length × width^2 ^× 0.5. The nude mice were sacrificed finally and the xenograft tumors were collected.

### Real-time PCR (RT-PCR)

Total RNA samples were extracted with TRIzol® reagent (Invitrogen; Thermo Fisher Scientific, Inc.). Reverse transcription was processed with the PrimeScript® RT Reagent kit (Takara Biotechnology Co., Ltd., Dalian, China) according to the instructions. In order to quantify the gene expression, SYBR Premix Ex Taq II (Takara Biotechnology Co., Ltd.) was utilized following the manufacturer’s protocol to conduct RT-PCR and the data were analyzed with 2^−ΔΔCq^ method. The primers for RT-PCR were listed in Supplementary Table 1.

### Nuclear extract preparation and western blotting analysis

The cells were collected and then lysed with RIPA buffer containing protease and phosphatase inhibitors. Nuclear Extraction Kit (Abcam, Cambridge, MA, USA) was utilized to acquire nuclear protein as described previously [21]. Protein quantification was performed with BCA method (Pierce; Thermo Fisher Scientific, Inc.). Proteins were separated by SDS-PAGE and then transferred to PVDF membranes. After blocking with skimmed milk, the membranes were incubated with various primary antibodies overnight at 4 °C, followed by HRP-conjugated secondary antibodies. Chemiluminescent HRP Substrate (Millipore, Billerica, MA, USA) and Western-Blot Imaging System were applied to visualize the protein bands. The gray level of the blots was quantified with Image software (National Institutes of Health, Bethesda, MD, USA). Antibodies used in the present study were displayed in Supplementary Table 2. Each experiment was repeated three times.

### Luciferase reporter assay

The MMP1 full promoter-reporter construct and the truncated ones were generated by inserting pGL3.0 Basic Vector (Promega, Madison, WI, USA) with various fragments of MMP1 5′-flanking sequence (Supplementary Table 1). The MMP1 promoter-reporter plasmids and the pTK-RL ones were co-transfected into cells. The detailed protocol was carried out as described previously [22].

### Quantitative chromatin immunoprecipitation (qChIP)

EZ-ChIP Kit (Millipore, Bedford, MA, USA) was applied following the manufacturer’s manual [22]. Briefly, chromatin-protein mixture was precipitated with 5 μg anti-ETV4 antibody and 1 μg IgG negative control antibody. Subsequently, they were amplified RT-PCR with specific primers corresponding to the target fragment or endogenous non-coding region fragment (Supplementary Table 1). The formula *E*^(Input Cq-ChIP Cq)^/*E*^(Input Cq-Control Cq)^ is adopted to estimate the enrichment index. Each experiment was repeated three times.

### Statistical analysis

All data are recorded as mean ± standard deviation (SD). Statistical significance between groups was evaluated appropriately by using Student’s *t*-test or one-way ANOVA, and *P* < 0.05 was considered as statistically significant. SPSS 18.0 software (SPSS Inc., Chicago, IL, USA) was used to perform statistical analysis. All in vitro experiments were done at least three times.

## Results

### TAMs enhanced the proliferation of colon cancer cells

**Fig. 1:**
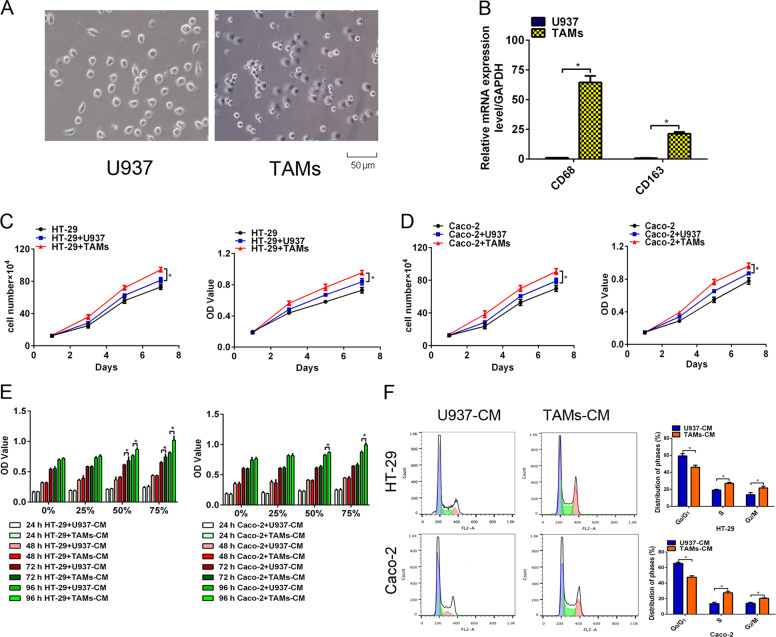
TAMs enhanced the proliferation of colon cancer cells. **A** After incubation with PMA and IL-4, the smooth surface morphology of U937 cells had a thorny appearance because of pseudopodia, and the suspended cells became adherent. **B** Comparison of the mRNA levels of CD68 and CD163 between U937 cells and TAMs by RT-PCR. **C**, **D** Cell growth and cell viability assays showed that co-culture with TAMs facilitated the growth and viability of HT-29 (**C**) and Caco-2 (**D**) cells in relation to the co-culture with U937 cells. **E** HT-29 and Caco-2 cells were cultured with 0, 25, 50, and 75% TAMs-CM or U937-CM for different time. Treatment with 50 or 75% TAMs-CM for 72 or 96 h promoted HT-29 and Caco-2 cells viability. **F** FACS analysis of cell cycle distribution indicated that treatment with TAMs-CM accelerated cell cycle transition from G0/G1 to S and G2/M phase. All data are presented as the mean ± SD from three independent experiments. *P < 0.05.

For activated macrophage (TAMs) induction, U937 cells were treated with PMA (10 ng/mL) and IL-4 (10 ng/mL) (Fig. 1A). The markers detected by RT-PCR indicated that CD68 and CD163 were elevated, which confirmed that the monocytes transformed into M2-type macrophages (Fig. 1B). Cell growth and cell viability assays showed that co-culture with TAMs facilitated the growth and viability of HT-29 and Caco-2 cells in relation to the co-culture with U937 cells (Fig. 1C, D). Then we treated HT-29 and Caco-2 cells with 0, 25, 50, and 75% TAMs conditioned media (TAMs-CM) or U937-CM for different time. HT-29 and Caco-2 cells treated with 50 or 75% TAMs-CM for 72 or 96 h showed more accelerated proliferative ability than their respective controls (Fig. 1E). Furthermore, we isolated macrophages from normal and paired CRC tissues. Flow cytometry screening was conducted to isolate CD68+ macrophages. After coculturing with TAMs from CRC tissues, HT-29 and Caco-2 cells showed enhanced capabilities of proliferation (Supplementary Fig. 1a and b). Treatment with TAMs-CM from CRC tissues with 50% or 75% TAMs-CM for 72 or 96 h displayed a similar alteration (Supplementary Fig. 1c).

Generally, the modulation of the cell cycle is involved in cell proliferation [23]. We attempted to assess the effect of TAMs on the cell cycle distribution of colon cancer cells by flow cytometry assay. HT-29 and Caco-2 cells cultured with TAMs-CM showed less percentage of cells in G0/G1 phase and more in S and G2/M phase (Fig. 1F). In addition, an accelerative cell cycle transition was observed in the TAMs-CM from CRC tissues coculture group compared with the control group (Supplementary Fig. 1d). From these observations, we concluded that TAMs accelerate the proliferation of colon cancer cells by accelerating the cell cycle transition from G0/G1 to S and G2/M phase.

### MMP1 contributed to the proliferation of colon cancer cells by accelerating the cell cycle transition from G0/G1 to S and G2/M phase

**Fig. 2:**
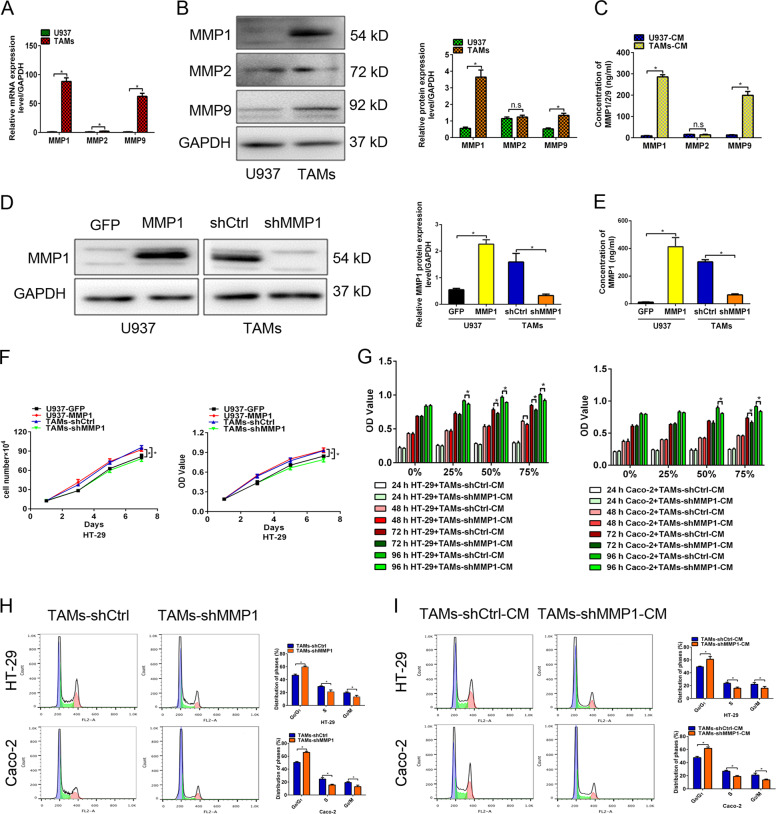
MMP1 contributed to the proliferation of colon cancer cells by accelerating the cell cycle transition from G0/G1 to S and G2/M phase. **A** Comparison of the mRNA levels of MMP1/2/9 between U937 cells and TAMs by RT-PCR. **B** Comparison of the protein levels of MMP1/2/9 between U937 cells and TAMs by western blotting analysis. **C** The release of MMP1/2/9 in U937 cells and TAMs detected by ELISA assay. **D** Western blotting analysis for MMP1 in MMP1-overexpressing U937 cells or MMP1-knockdown TAMs. **E** The release of MMP1 in MMP1-overexpressing U937 cells or MMP1-knockdown TAMs detected by ELISA assay. **F** The effect of co-culture with TAM-shMMP1 or U937-MMP1 on the growth and viability of HT-29 cells. **G** HT-29 and Caco-2 cells were treated with 0, 25, 50, and 75% TAMs- shMMP1-CM for different time. The effect of treatment with TAMs-shMMP1-CM on the viability of HT-29 and Caco-2 cells was detected by cell viability assays. **H**, **I** The effect of co-culture with TAMs-shMMP1 (**H**) or treatment with TAMs- shMMP1-CM (**I**) on the cell cycle distribution of HT-29 and Caco-2 cells detected by FACS analysis. All data are presented as the mean ± SD from three independent experiments. *P < 0.05.

Then we detected the expression of several soluble factors between U937 and TAMs. An elevated level of MMP1/9, especially MMP1, was observed in TAMs by RT-PCR and western blotting analysis (Fig. 2A, B). Furthermore, the result from ELISA assay confirmed that the concentration of MMP1/9 in TAMs-CM was significantly higher than that in U937-CM (Fig. 2C). We further examined the cytokines produced by patient-derived TAMs. As expected, the results showed that CD163+ M2 macrophages highly expressed MMP1 (Supplementary Fig. 1e). Thus, we inferred that TAMs facilitate the proliferation of colon cancer cells, which may be attributed to the paracrine factor including MMP1 secreted by TAMs.

To explore the role of MMP1 in the proliferation of colon cancer cells facilitated by TAMs, we constructed MMP1-shRNA or MMP1-overexpressing lentivirus and transfected them into TAMs or U937 cells, respectively. Knockdown of MMP1 in TAMs (TAMs-shMMP1) and ectopic expression of MMP1 in U937 cells (U937-MMP1) were validated by western blotting analysis (Fig. 2D). Moreover, the release of MMP1 is reduced in TAMs-shMMP1 but elevated in U937-MMP1 compare to their respective control (Fig. 2E).

As expected, co-culture with TAMs-shMMP1 results in a proliferation inhibition of HT-29 and Caco-2 cells, compared to co-culture with TAMs-shCtrl (Fig. 2F; Supplementary Fig. 2a). Similarly, HT-29 and Caco-2 cells treated with 50 or 75% TAMs-shMMP1-CM for 72 or 96 h showed reduced proliferative ability (Fig. 2G). However, co-culture with U937-MMP1 or treatment with U937-MMP1-CM had the opposite effects (Supplementary Fig. 2a, b). We further treated HT-29 and Caco-2 cells with TAMs from CRC tissues combining MMP1 neutralizing antibodies (Abs). Consistently, MMP1 neutralizing Ab treatment suppressed the proliferation induced by TAMs from CRC tissues (Supplementary Fig. 1f, g). In addition, recombinant human MMP1 (rhMMP1) was applied to the media of HT-29 and Caco-2 cells. Treatment with rhMMP1 facilitated the proliferation of HT-29 and Caco-2 cells in a dose- and time-dependent manner (Supplementary Fig. 2c–e).

The impact of exogenous MMP1 on cell cycle distribution was evaluated by flow cytometry assay. Co-culture with TAMs-shMMP1 resulted in an increased percentage of cells in G0/G1 phase and a decreased percentage in S and G2/M phase compared to the control (Fig. 2H). Similar results were observed in HT-29 and Caco-2 cells with TAMs-shMMP1-CM (Fig. 2I). Conversely, co-culture with U937-MMP1 or treatment with U937-MMP1-CM or rhMMP1 reduced the proportion of cells in the G0/G1 phase and elevated the proportion in the S and G2/M phase (Supplementary Fig. 2f–h). In addition, MMP1 neutralizing Abs (NAs) treatment retarded cell cycle transition promoted by TAMs from CRC tissues (Supplementary Fig. 1h). Altogether, these data indicated that exogenous MMP1 secreted by TAMs contributed to the proliferation of colon cancer cells by accelerating the cell cycle transition from G0/G1 to S and G2/M phase.

### MMP1 altered the expression of cell cycle-related gene through c-Myc and ETV4

**Fig. 3:**
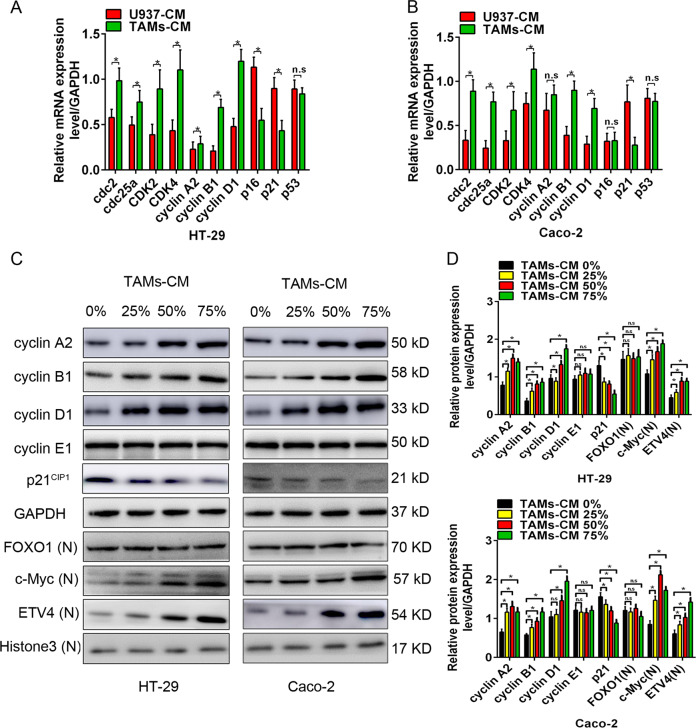
MMP1 altered the expression of cell cycle-related gene through c-Myc and ETV4. **A**, **B** The effect of co-culture with TAMs-CM on the mRNA levels of cell cycle-related gene in HT-29 (**A**) and Caco-2 (**B**) cells detected by RT-PCR. **C** Western blotting analysis for cell cycle-related proteins and c-Myc and ETV4 in HT-29 and Caco-2 cells with 0, 25, 50, and 75% TAMs-CM. **D** Comparison of the protein levels in HT-29 and Caco-2 cells. All data are presented as the mean ± SD from three independent experiments. *P < 0.05.

To further elucidate the potential mechanism of MMP1-induced proliferation of colon cancer cells, we examined the expression of several cell cycle-related genes by RT-PCR and western blotting analysis. The result from RT-PCR showed that TAMs-CM incubation or rhMMP1 treatment increased the mRNA levels of cdc2, cdc25a, CDK2, CDK4, cyclin B1, and cyclin D1 but reduced the level of p21 (Fig. 3A, B; Supplementary Fig. 3a, b). Consistent with the alterations of these genes at mRNA level, TAMs-CM incubation or rhMMP1 treatment dose-dependently elevated the protein levels of cyclin A2, cyclin B1, and cyclin D1 but decreased the level of p21 (Fig. 3C, D; Supplementary Fig. 3c, d).

FOXO1, c-Myc, and ETV4 are three classical transcription factors that modulate cell cycle via targeting p21, cyclinA2, cyclinB1, and cyclinD1, among others [24–26]. Our study revealed an elevated protein level of c-Myc and ETV4 in HT-29 and Caco-2 cells with TAMs-CM incubation (Fig. 3C, D) or rhMMP1 treatment (Supplementary Fig. 3c, d). However, no significant alteration of FOXO1 was observed. Combined with these findings, we speculated that MMP1 might accelerate the cell cycle transition of colon cancer cells by regulating cdc25a/CDK4-cyclin D1 and p21/cdc2-cyclin B1 complexes through alteration in the expression of c-Myc and ETV4.

### MMP1 facilitated the proliferation of colon cancer cells by activating PAR1

Notably, PAR1 has been identified as a receptor of MMP1 [15]. To determine the role of PAR1 in MMP1-induced proliferation of colon cancer cells, PAR1 inhibitor MK-5348 or knockdown of PAR1 was performed in our study. Cell growth and cell viability assays showed that treatment with MK-5348 eliminated the proliferation of HT-29 and Caco-2 cells accelerated by TAMs-CM incubation or rhMMP1 treatment (Supplementary Fig. 4a–d). Knockdown of PAR1 had a similar effect (Supplementary Fig. 4e–h). Consistently, the alteration of cell cycle-related proteins induced by exogenous MMP1 was abrogated upon MK-5348 treatment (Supplementary Figs. 5c, d and 6c, d). These findings indicated that MMP1 contributes to the proliferation of colon cancer cells and cell cycle transition by activating its receptor PAR1.

### The MMP1/PAR1 axis facilitated colon cancer cell proliferation via the phosphorylation of Erk1/2

**Fig. 4:**
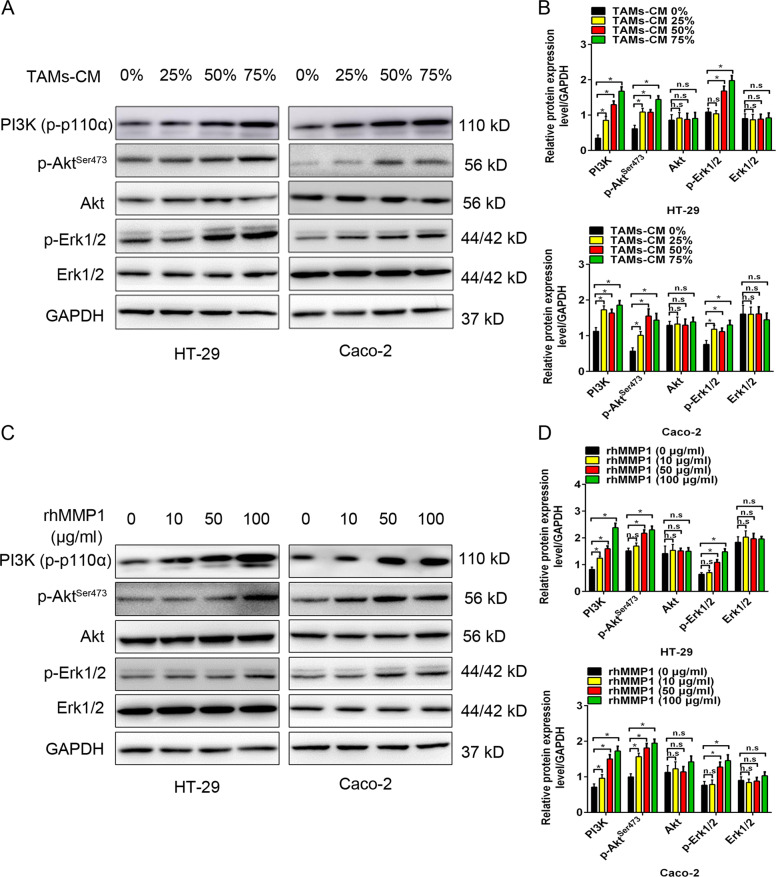
MMP1 promoted the phosphorylation of Erk1/2 and Akt. **A**, **C** Western blotting analysis for PI3K/Akt and MAPK/Erk signaling in HT-29 and Caco-2 cells treated with 0, 25, 50, and 75% TAMs-CM (**A**) or 0, 10, 50, 100 μg/ml rhMMP1 (**C**). **B**, **D** Comparison of the protein levels in HT-29 and Caco-2 cells treated with 0, 25, 50, and 75% TAMs-CM (**B**) or 0, 10, 50, 100 μg/ml rhMMP1 (**D**). All data are presented as the mean ± SD from three independent experiments. *P < 0.05.

It is well known that c-Myc and ETV4 are regulated by Akt and Erk1/2 downstream of PI3K/Akt and MAPK/Erk pathways [27, 28]. Hence, our study detected the effect of exogenous MMP1 on the activity of PI3K/Akt and MAPK/Erk pathways. HT-29 and Caco-2 cells treated with TAMs-CM incubation showed an elevated level of PI3K (p-p100α), p-Akt (S473), and p-Erk1/2 (T202/Y204) (Fig. 4A, B). Similar results were observed in HT-29 and Caco-2 cells with rhMMP1 treatment (Fig. 4C, D).

To further determine that PI3K/Akt and MAPK/Erk pathways were responsible for MMP1/PAR1 axis-induced proliferation of colon cancer cells, an Erk1/2 inhibitor (SCH772984) and an Akt inhibitor (MK-2206) were applied in the present study. Treatment with SCH772984 but not MK-2206 suppressed the accelerative proliferation of HT-29 and Caco-2 cells induced by TAMs-CM incubation or rhMMP1 treatment (Supplementary Figs. 5a, b and 6a, b). Western blotting analysis revealed that SCH772984 treatment reduced the protein levels of cyclin A2, cyclin B1, cyclin D1, c-Myc, and ETV4 but elevated the protein levels of p21 (Supplementary Figs. 5c, d and 6c, d). No significant alteration of cell cycle-related proteins was observed upon MK-2206 treatment. Likewise, treatment with PAR1 inhibitor MK-5348 inhibited the expression of p-Erk1/2 but did not affect p-Akt (Supplementary Figs. 5c, d and 6c, d). Altogether, these findings suggested that MMP1/PAR1 axis facilitates the proliferation of colon cancer cells through MAPK/Erk signaling.

### The positive feedback between MMP1 and ETV4 exists in colon cancer cells

**Fig. 5:**
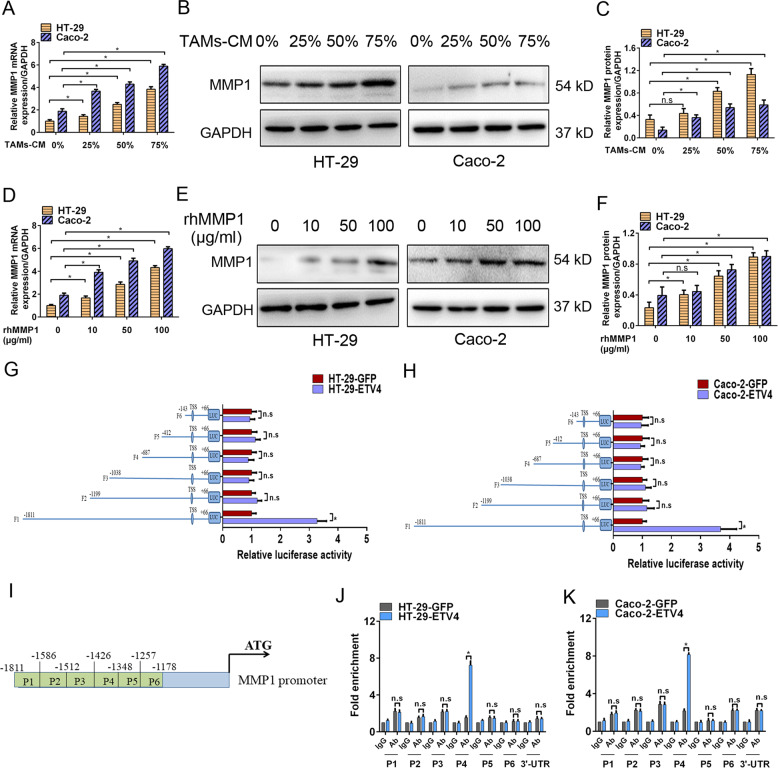
The positive feedback between MMP1 and ETV4 exists in colon cancer cells. **A**, **D** Comparison of the mRNA level of MMP1 in HT-29 and Caco-2 cells treated with 0, 25, 50, and 75% TAMs-CM (**A**) or 0, 10, 50, and 100 μg/ml rhMMP1 (**D**) detected by RT-PCR. **B**, **E** Western blotting analysis for MMP1 in HT-29 and Caco-2 cells treated with 0, 25, 50, and 75% TAMs-CM (**B**) or 0, 10, 50, and 100 μg/ml rhMMP1 (**E**). **C**, **F** Comparison of the protein levels in HT-29 and Caco-2 cells. **G**, **H** The activities of the MMP1 full promoter-reporter construct and the truncated ones in ETV4-overexpressing HT-29 (**G**) and Caco-2 (**H**) cells using the dual-luciferase assay. **I** Schematic representation of the ETV4 putative binding sites (P1-6) in MMP1 promoter region. **J**, **K** Enrichment level of ETV4-binding putative site in the MMP1 promoter region in HT-29 (**J**) and Caco-2 (**K**) cells determined by qChIP assay. All data are presented as the mean ± SD from three independent experiments. *P < 0.05.

Intriguingly, HT-29 and Caco-2 cells with TAMs-CM incubation or rhMMP1 treatment displayed an elevated expression of MMP1 at both mRNA and protein levels (Fig. 5A–F). We speculated that there might exist a positive feedback loop regulating MMP1 expression. By using bioinformatics analysis, several potential ETV4-binding sites (AGGAAG/AT) were identified in the MMP1 promoter. A dual-luciferase reporter assay was then performed to determine that whether ETV4 transcriptionally activates MMP1 expression. First of all, the full-length MMP1 promoter (from −1811 bp to +66 bp) reporter construct and the other three truncated ones were constructed and transfected into HT-29 and Caco-2 cells, respectively. Ectopic expression of ETV4 elevated the luciferase activity of the full-length fragment but did not affect other truncated fragments (Fig. 5G, H), suggesting that ETV4 could trans-activate MMP1 expression by binding to the −1811 bp to −1200 bp of the MMP1 promoter.

Next, we attempted to confirm that whether ETV4 protein binds to the special site of the MMP1 promoter in vivo by using qChIP assay. Six pairs of primers were designed to amplify the four P1-P6 fragments of the −1811 bp to −1200 bp of MMP1 promoter region (Fig. 5I). The results showed that ectopic expression of ETV4 enhanced the binding of ETV4 to P4 promoter fragment but not the other promoter fragments (Fig. 5J, K). All of these results indicated that ETV4 could bind to the P4 fragment of the MMP1 promoter and transcriptionally activate MMP1 in colon cancer cells, which confirmed the MMP1/ETV4/MMP1 positive feedback.

### Essential role of MMP1/PAR1/Erk1/2 axis in tumor formation of colon cancer cells

**Fig. 6:**
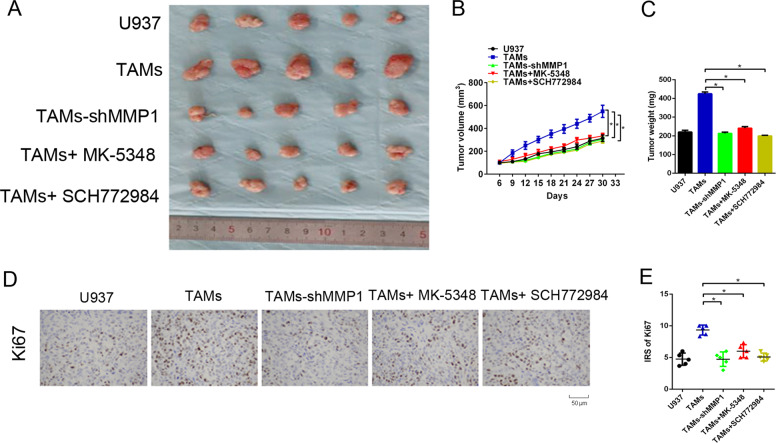
Essential role of MMP1/PAR1/Erk1/2 axis in tumor formation of colon cancer cells. **A** Schematic representation of the tumor xenografts formed by TAMs and HT-29 cells cells with PAR1 inhibitor MK-5348 or Erk1/2 inhibitor SCH772984. **B**, **C** Tumor growth curves (**B**) and tumor weights (**C**) for tumor xenograft formed by TAMs and HT-29 cells cells with MK-5348 or SCH772984. **D** IHC staining for Ki67 in tumor xenografts formed by TAMs and HT-29 cells cells with MK-5348 or SCH772984. **E** Comparison of immunoreactivity scores (IRS) of Ki67 among the five group. *P < 0.05.

To further evaluate the role of MMP1/PAR1/Erk1/2 axis in the tumor formation ability of colon cancer cells in vivo, we injected TAMs+HT-29 cells into BALB/c mice to establish a model of subcutaneous xenografts. Once palpable xenograft tumors were established, the nude mice were intraperitoneally injected with PAR1 inhibitor MK-5348 or Erk1/2 inhibitor SCH772984. The xenograft tumors formed by TAMs-shMMP1+HT-29 developed much more slowly and were lighter than those formed by TAMs+HT-29 cells (Fig. 6A–C). Treatment with MK-5348 or SCH772984 had similar results. Mechanistically, the xenograft tumors formed by TAMs-shMMP1+HT-29 cells showed a much weaker Ki67 staining score than those formed by TAMs+HT-29 cells (Fig. 6D, E). Similar results were observed in xenograft tumors upon MK-5348 or SCH772984 treatment. These data further demonstrated that MMP1/PAR1/Erk1/2 axis promotes tumor formation of colon cancer cells.

## Discussion

In recent decades, TME attracts more attention for its critical role in tumor development and progression [6]. Elucidating the molecular mechanism involved might highlight better therapeutic strategies against cancer. In the present study, we demonstrated that co-culture with TAMs accelerated the proliferation of HT-29 and Caco-2 cells. Further investigation revealed that TAMs highly expressed MMP1 and MMP9 at both mRNA and protein levels in relation to U937, which was further confirmed by ELISA assay, in which the level of MMP1 released by TAMs was more than that released by U937. Similar alterations were observed by Shankar et al. in the secretomes of macrophages [29]. TAMs contribute to tumor progression and invasion by secreting MMPs [30, 31]. The level of MMP1 strongly correlates with advanced colon cancer, metastatic dissemination, and an adverse outcome [32, 33].

Mechanistically, treatment with rhMMP1 or TAMs-CM promoted the proliferation and cell cycle transition from G0/G1 to S and G2/M phases of colon cancer cells, which mimic the proliferation-stimulating effect of co-culture with TAMs. Among cell cycle-related genes that we detested, the expression levels of cdc2, cdc25a, CDK2, CDK4, cyclin A2, cyclin B1, and cyclin D1 were elevated, and the expression level of p21 was reduced in HT-29 and Caco-2 with rhMMP1 treatment or TAMs-CM incubation, suggesting that MMP1 promoted cell cycle acceleration of colon cancer cells by activating cdc25a/CDK4-cyclin D1 and p21/cdc2-cyclin B1 complexes. Liu et al. reported that MMP1 promoted tumor growth by regulating c-Myc expression [34]. Our data indicated that alterations in cell cycle transition and the expression of cell cycle-related proteins triggered by MMP1 might be related to the up-regulation of c-Myc and ETV4. In addition, MMP1 contributes to the metastatic phenotype of colon cancer cells [35].

ETV4 has been previously shown to regulate MMP1 expression, despite that the molecular mechanism involved remains elusive [36]. Intriguingly, the current study indicated that HT-29 and Caco-2 cells with rhMMP1 treatment or TAMs-CM incubation displayed an elevated level of MMP1. Mechanistically, our study demonstrated that ETV4 specifically and directly binds to the MMP1 promoter and promotes MMP1 transcription by the dual-luciferase reporter and qChIP assays. Collectively, the MMP1/ETV4/MMP1 positive feedback in CRC was for the first time confirmed in our study.

A newly identified mechanism of MMP1 in tumor promotion is through activation of oncogenic signaling pathway downstream of PAR1 cleavage [20, 34]. Treatment with PAR1 inhibitor MK-5348 or knockdown of PAR1 abolished the proliferation of HT-29 and Caco-2 cells stimulated by rhMMP1 treatment or TAMs-CM incubation. Our study for the first time revealed MMP1 derived from TAMs can activate tumor-expressed PAR1 and thereby facilitate the proliferation of colon cancer cells. Pharmacologic blockade of PAR1 has been shown to repress tumor survival, angiogenesis, and metastasis [37, 38], which is expected to represent a promising therapeutic strategy against cancer [39]. Three PAR1 antagonists (vorapaxar, atopaxar, and PZ-128) have undergone clinical trial at various stages for the treatment of cardiovascular diseases. The combination of traditional chemotherapy (e.g., 5-Fu and doxycycline) plus PZ-128 elicited synergistic tumor inhibition in pancreatic and breast cancer [40]. However, given that PAR1 was orchestrated by a diverse set of unique proteases canonical and noncanonical (e.g., MMP1 or thrombin), targeting PAR1 might trigger diverse downstream signaling due to diverse allosteric conformations, hindering the development of PAR1 inhibitor [39].

The notion of MMP1/PAR1 axis was initially demonstrated in the process of infection [41]. Later, emerging evidence indicated that MMP1/PAR1 axis participated in the pathogenesis of thrombosis [42], atherosclerosis [43], and serious cardiac events [44]. Recent studies support a critical role of MMP1/PAR1 axis in tumor development and progression. MMP1 and PAR1 were co-expressed in gall bladder cancer [18], breast cancer [16], prostate cancer [45], and liver cancer [17]. Stromal MMP1 stimulated the aggressive behavior of breast cancer cells through PAR1 to promote tumor progression [16]. Moreover, MMP1/PAR1 axis contributed to the perineural invasion (PNI) of pancreatic cancer cells [20]. MMP1/PAR1 activation induced the secretion of angiogenic factors (eg. IL-18, MCP-1, and GRO-α) in ovarian cancer [46].

MAPK/Erk and PI3K/Akt pathway were newly identified as the mechanism of MMP1 in tumor promotion [20, 47]. The result from our study showed that both MAPK/Erk and PI3K/Akt pathway were significantly activated by rhMMP1 treatment or TAMs-CM incubation. Intriguingly, PAR1 inhibitor MK-5348 repressed the activity of MAPK/Erk pathway but not PI3K/Akt pathway. Furthermore, blockage of MAPK/Erk pathway by Erk1/2 inhibitor SCH772984 eliminated the proliferation of colon cancer cells induced by rhMMP1 treatment or TAMs-CM incubation. Correspondingly, the levels of cyclin A2, cyclin B1, cyclin D1, c-Myc, and ETV4 were downregulated, and the expression level of p21 was upregulated in response to Erk1/2 inhibitor treatment. However, blockage of PI3K/Akt pathway by AKT inhibitor MK-2206 did not affect the tumor-promoting effect of exogenous MMP1 on colon cancer cells. The vivo results of our study showed that inhibition of PAR1 or blockage of MAPK/Erk pathway attenuated the growth rates and the weights of xenograft tumors. Based on these observations, we concluded that MMP1/PAR1 axis facilitates colon cancer cell proliferation through MAPK/Erk pathway. Consistently, blocking PAR1 signaling repressed activation of Erk1/2 in gastric cancer [48]. However, MMP1/PAR1 axis was reported to activate PI3K/AKT pathway in pancreatic cancer [20]. Upon PAR1 overexpression, persistent activation of Akt was elicited in breast cancer both in vivo and in vitro [37]. In addition, PAR1 regulates proliferative and migratory responses through FAK and Smad2 [49]. Altogether, these data more definitively establish MMP1 as a signaling molecule that can elicit direct and diverse cellular effects to accelerate tumor progression, and further expand the repertoire of MMP functions.

In conclusion, our study for the first time reveals that MMP1 derived from TAMs activates MAPK/Erk signaling pathway through paracrine PAR1 activation, which affects the expression of cell cycle-related gene, including cdc25a/CDK4-cyclin D1 and p21/cdc2-cyclin B1 complexes and ultimately facilitates colon cancer cells proliferation.

## Supplementary information

Supplementary figure 1

Supplementary figure 2

Supplementary figure 3

Supplementary figure 4

Supplementary figure 5

Supplementary figure 6

Supplementary figure legend

Supplementary table

## Data Availability

The datasets used and/or analyzed during the current study are available from the corresponding authors on reasonable request.
