# Supplementary figures and images for: Tumor-associated macrophages (TAMs) depend on MMP1 for their cancer-promoting role
Source: Cell Death Discov. 2021 Nov 9;7:343. doi: 10.1038/s41420-021-00730-7 (PMC8578434; doi:10.1038/s41420-021-00730-7)

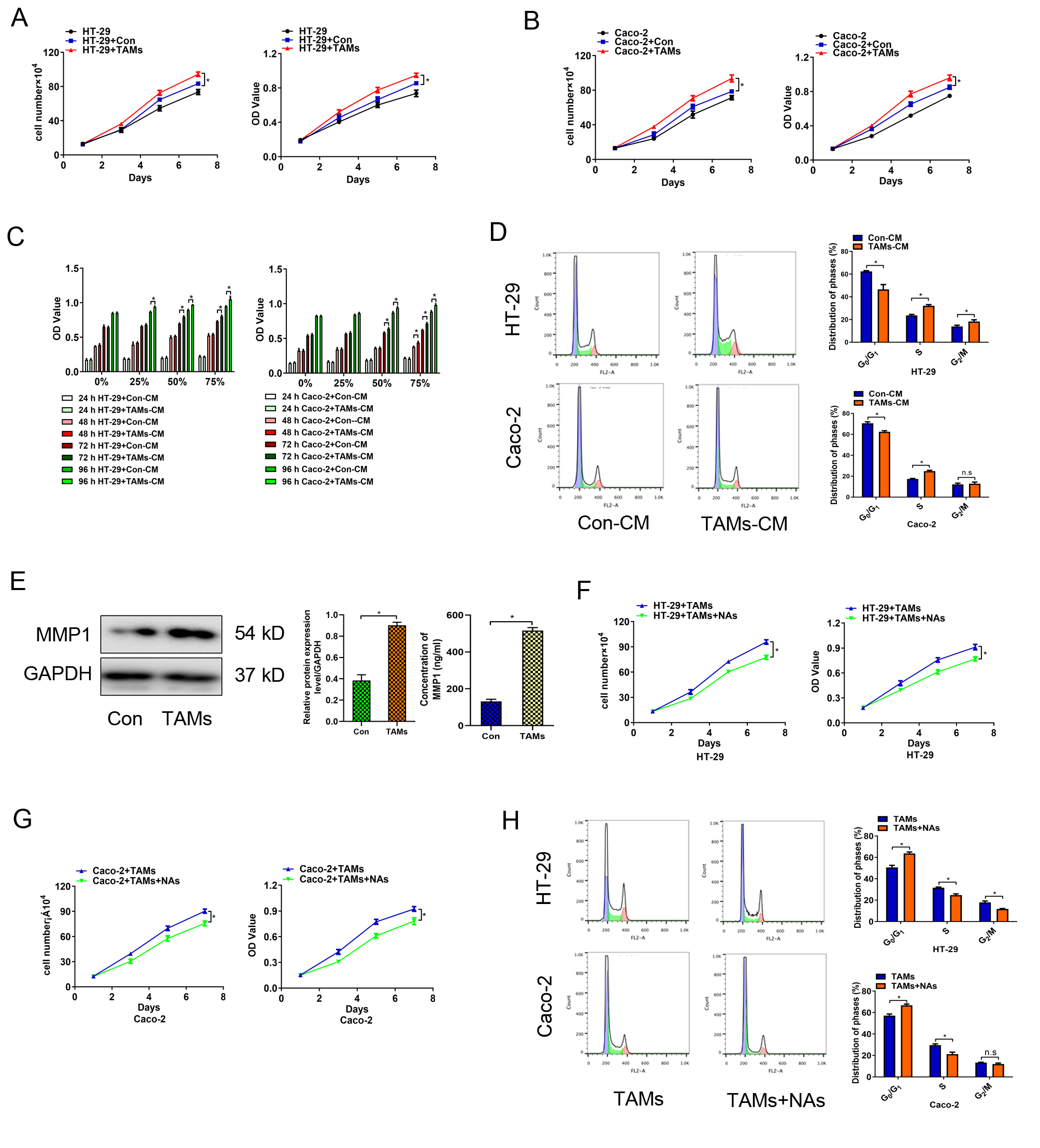

Supplement: Supplementary file 1 — Supplementary figure 1 [file 41420_2021_730_MOESM1_ESM.tif]

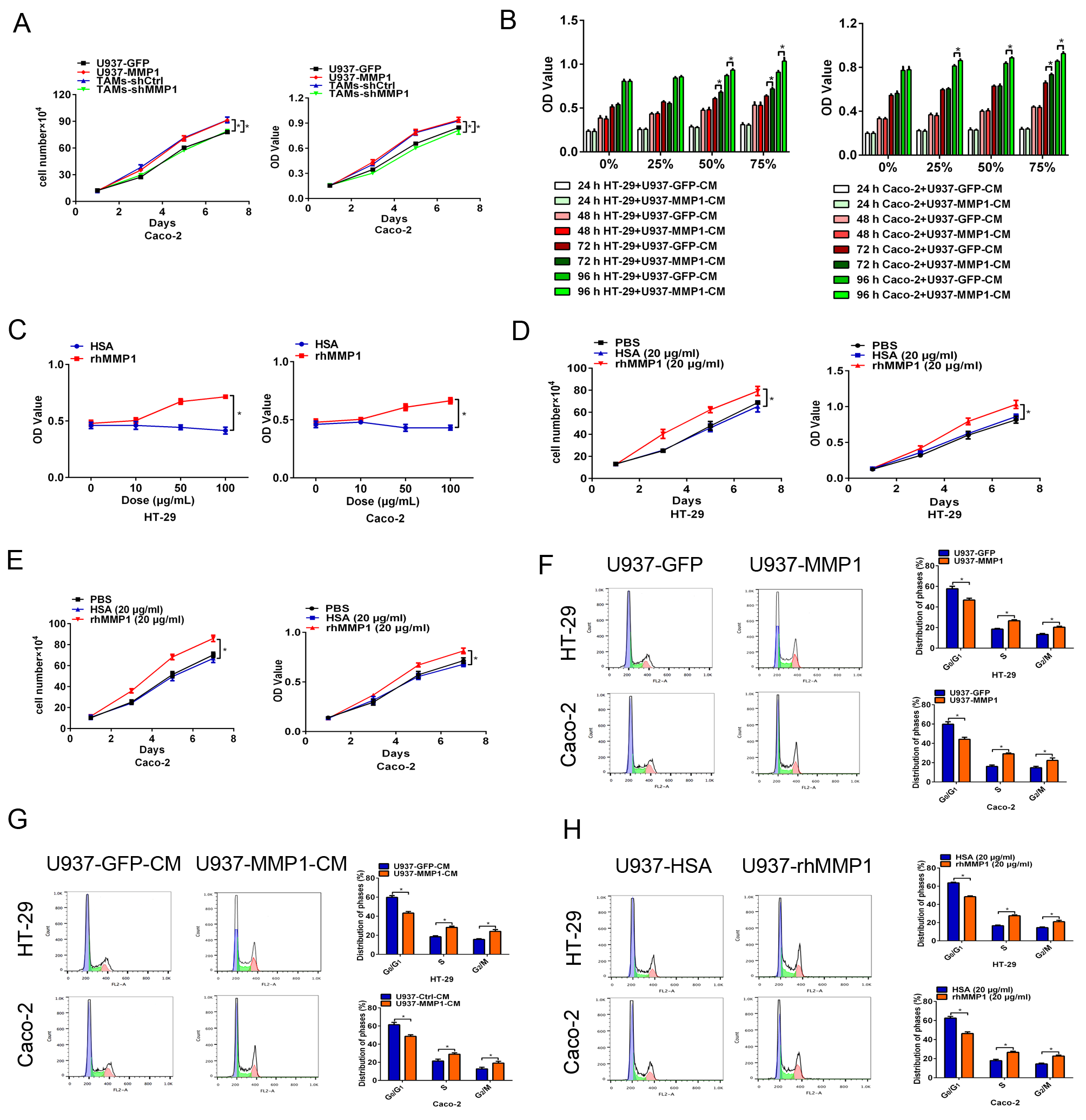

Supplement: Supplementary file 2 — Supplementary figure 2 [file 41420_2021_730_MOESM2_ESM.tif]

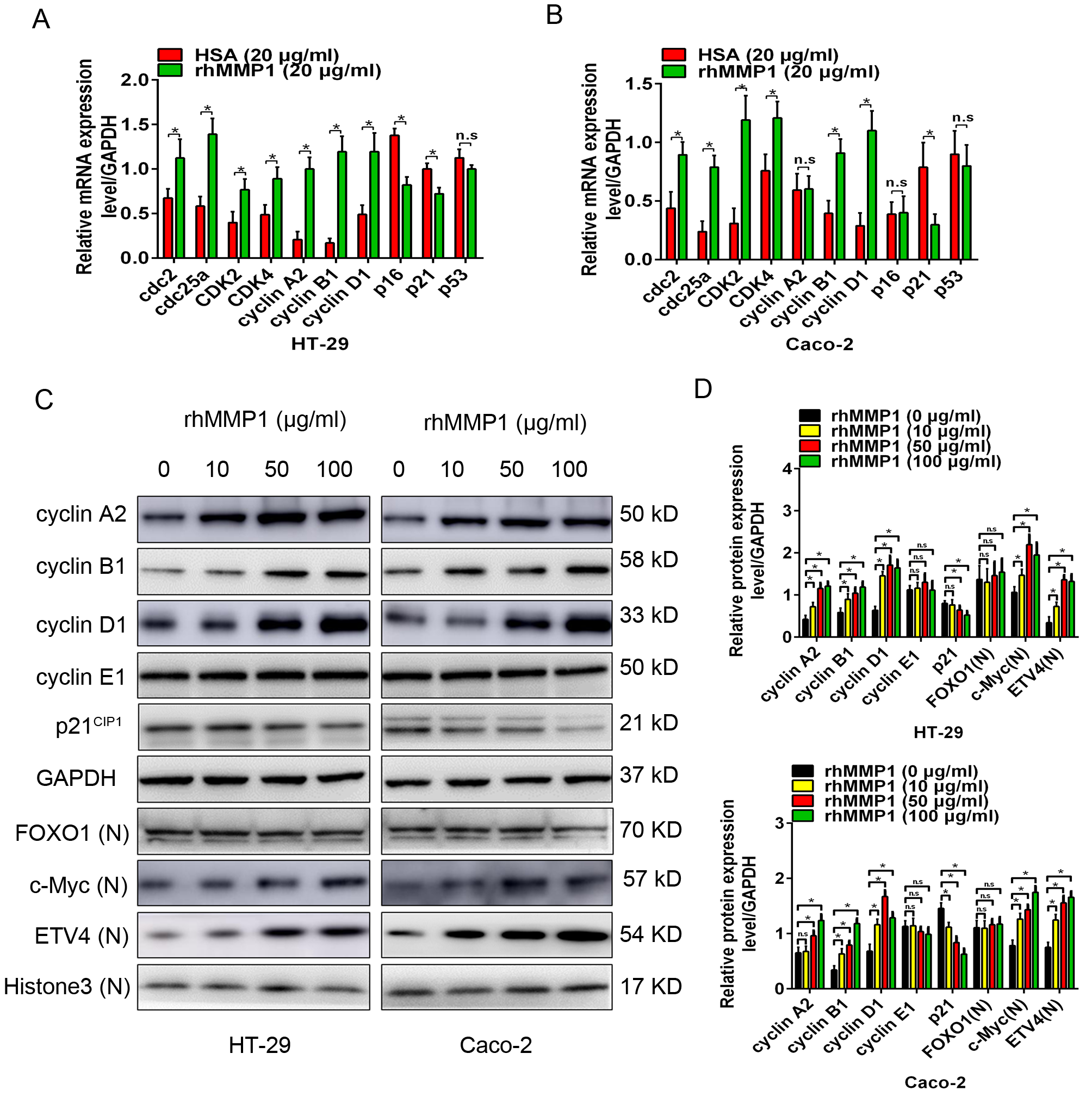

Supplement: Supplementary file 3 — Supplementary figure 3 [file 41420_2021_730_MOESM3_ESM.tif]

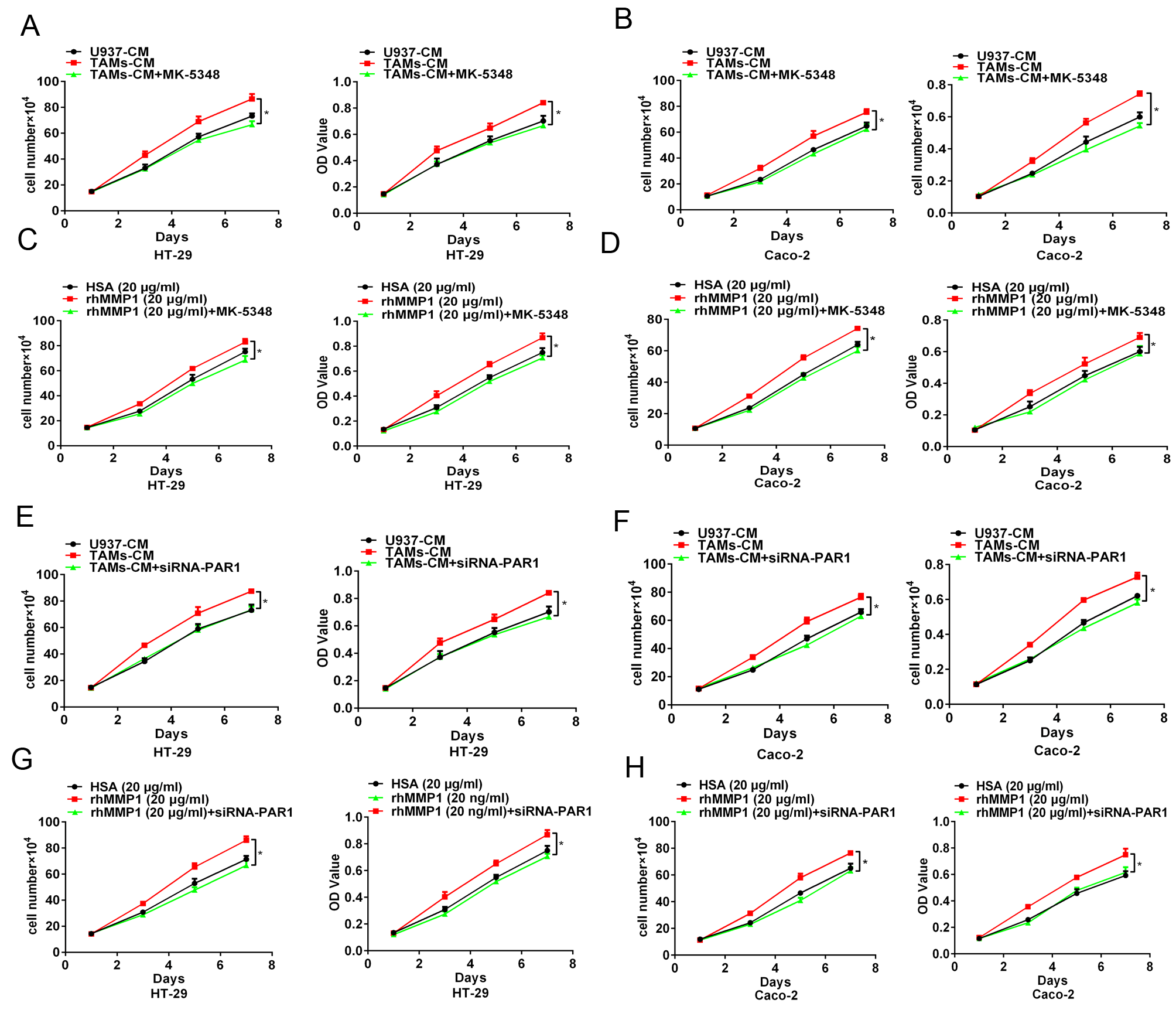

Supplement: Supplementary file 4 — Supplementary figure 4 [file 41420_2021_730_MOESM4_ESM.tif]

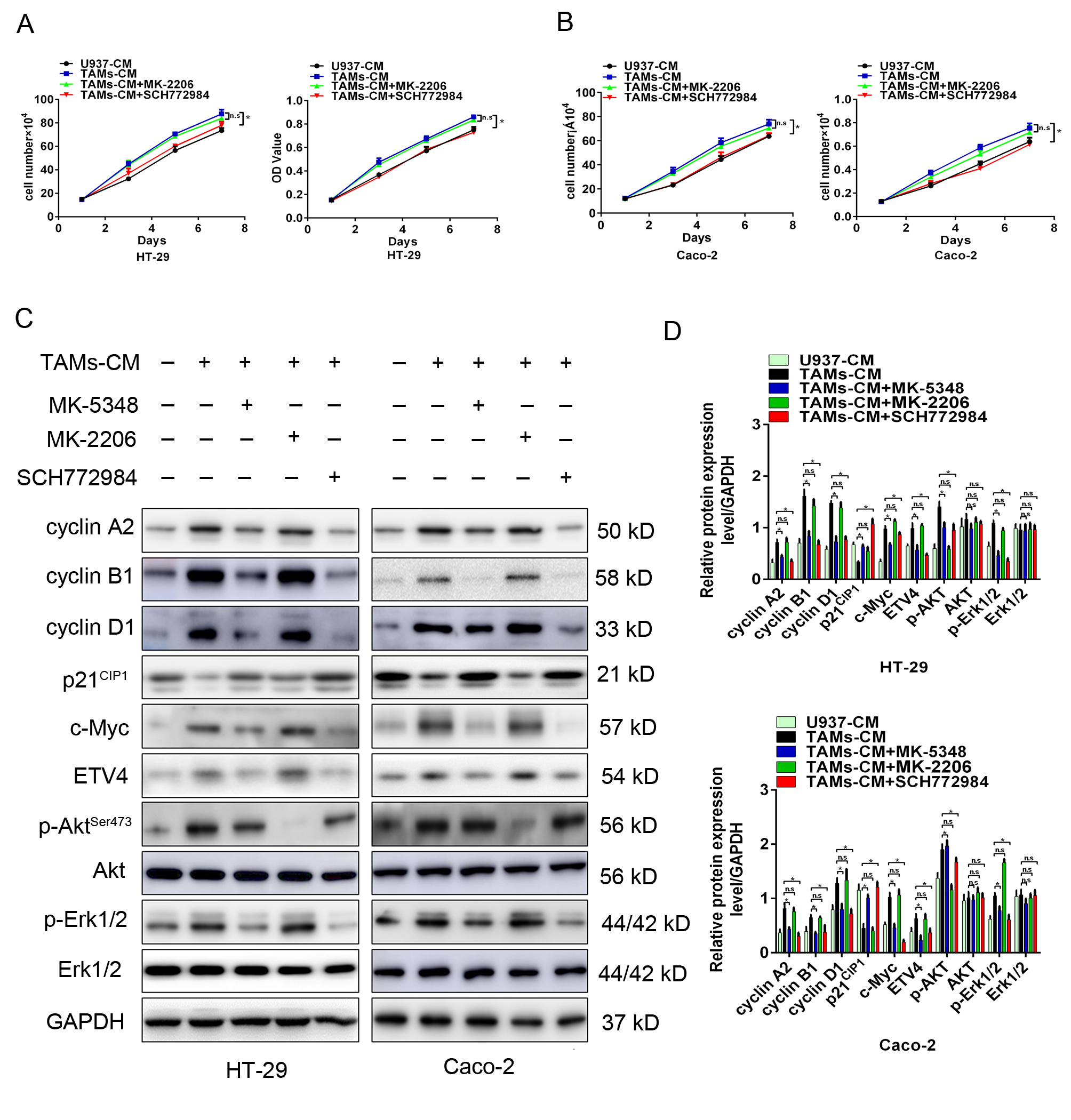

Supplement: Supplementary file 5 — Supplementary figure 5 [file 41420_2021_730_MOESM5_ESM.tif]

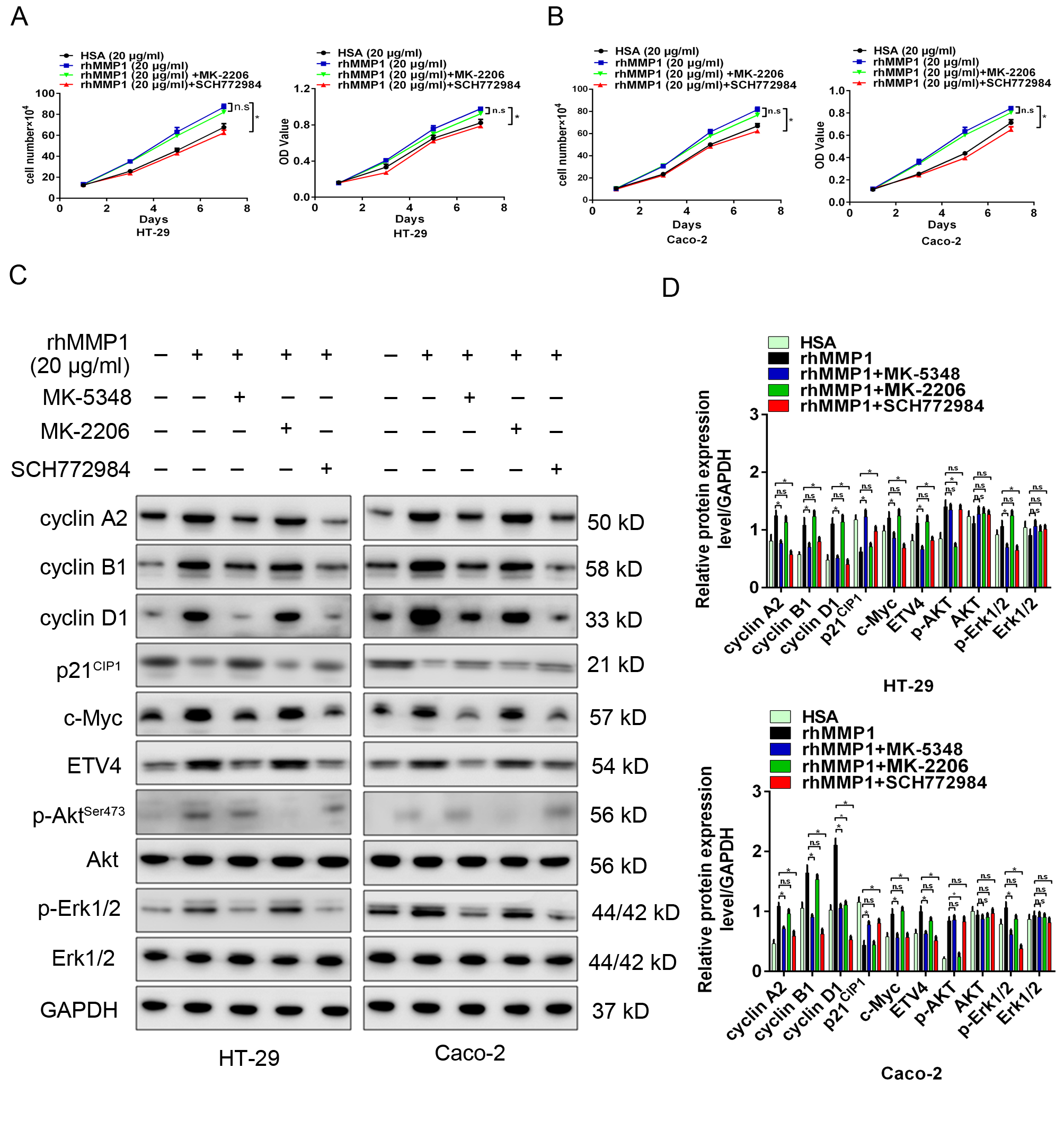

Supplement: Supplementary file 6 — Supplementary figure 6 [file 41420_2021_730_MOESM6_ESM.tif]
