# Supplementary material for: Tumor-associated macrophages (TAMs) depend on MMP1 for their cancer-promoting role
Source: Cell Death Discov. 2021 Nov 9;7:343. doi: 10.1038/s41420-021-00730-7 (PMC8578434; doi:10.1038/s41420-021-00730-7)
Supplement: Supplementary file 7 — Supplementary figure legend [file 41420_2021_730_MOESM7_ESM.docx]

**Supplementary figure 1 TAMs enhanced the** **proliferation of colon cancer cells depending on MMP1**

**a, b** Cell growth and cell viability assays showed that co-culture with TAMs from CRC tissues facilitated the growth and viability of HT-29 (**a**) and Caco-2 (**b**) cells in relation to the control. **c** HT-29 and Caco-2 cells were cultured with 0%, 25%, 50% and 75% TAMs-CM or U937-CM from CRC tissues for different time. Treatment with 50% or 75% TAMs-CM for 72 or 96 h promoted HT-29 and Caco-2 cells viability. **d** FACS analysis of cell cycle distribution indicated that treatment with TAMs-CM from CRC tissues accelerated cell cycle transition from G0/G1 to S. **e** Comparison of the protein levels of MMP1 between TAMs from CRC tissues and control cells by western blotting analysis and ELISA. **f, g** HT-29 and Caco-2 cells with TAMs from CRC tissues combining MMP1 neutralizing Abs (NAs). Cell growth and cell viability assays showed that MMP1 NAs treatment suppressed the proliferation of HT-29 (**f**) and Caco-2 (**g**) induced by TAMs from CRC tissues. **h** FACS analysis of cell cycle distribution indicated that MMP1 NAs treatment retarded cell cycle transition promoted by TAMs from CRC tissues. All data are presented as the mean±SD from three independent experiments. **P* <0.05.

**Supplementary figure 2 MMP1 contributed to the proliferation of colon cancer cells enhanced by TAMs a** The effect of co-culture with TAM-shMMP1 or U937-MMP1 on the growth and viability of Caco-2 cells. **b** HT-29 and Caco-2 cells were treated with 0%, 25%, 50% and 75% U937-MMP1-CM for different time. The effect of treatment with U937-MMP1-CM on the viability of HT-29 and Caco-2 cells detected by cell viability assays. **c** HT-29 and Caco-2 cells were treated with various doses of rhMMP1 (0, 10, 50 and 100 μg/ml) for 2 days. **d, e** HT-29 (**d**) and Caco-2 (**e**) cells were treated with 20 μg/ml rhMMP1 for different periods of time (1, 3, 5 and 7 days). **f-h** The effect of co-culture with U937-MMP1 (**f**) or treatment with U937-MMP1-CM (**g**) or rhMMP1 (**h**) on the cell cycle distribution of HT-29 and Caco-2 cells detected by FACS analysis. All data are presented as the mean±SD from three independent experiments. **P* <0.05.

**Supplementary figure 3** **MMP1 altered the expression of cell cycle-related genes through c-Myc and ETV4 a, b** The effect of treatment with rhMMP1 (0, 10, 50 and 100 μg/ml) on the mRNA levels of cell cycle-related gene in HT-29 (**a**) and Caco-2 (**b**) cells detected by RT-PCR. **c** Western blotting analysis for cell cycle-related proteins and c-Myc and ETV4 in HT-29 and Caco-2 cells treated with 0, 10, 50 and 100 μg/ml rhMMP1. **d** Comparison of the protein levels in HT-29 and Caco-2 cells. All data are presented as the mean±SD from three independent experiments. **P* <0.05.

**Supplementary figure 4 MMP1 facilitated the proliferation of colon cancer cells by activating PAR1 a, b** The effect of PAR1 inhibitor MK-5348 on the growth and viability of HT-29 (**a**) and Caco-2 (**b**) co-cultured with TAMs-CM. **c, d** The effect of MK-5348 on the growth and viability of HT-29 (**c**) and Caco-2 (**d**) cells treated with 20 μg/ml rhMMP1. **e, f** The effect of PAR1 knockdown by siRNA on the growth and viability of HT-29 (**e**) and Caco-2 (**f**) co-cultured with TAMs-CM. **g, h** The effect of PAR1 knockdown by siRNA on the growth and viability of HT-29 (**g**) and Caco-2 (**h**) cells treated with 20 μg/ml rhMMP1. All data are presented as the mean±SD from three independent experiments. **P* <0.05.

**Supplementary figure 5 Blockage of MAPK/Erk signaling eliminated the proliferation of colon cancer cells induced by TAMs-CM incubation a, b** The effect of Erk1/2 inhibitor (SCH772984) and an Akt inhibitor (MK-2206) on the growth and viability of HT-29 (**a**) and Caco-2 (**b**) cells treated with TAMs-CM. **c** Western blotting analysis for the effect of SCH772984, MK-2206 and MK-5348 on cell cycle-related proteins and c-Myc and ETV4 in HT-29 and Caco-2 cells treated with TAMs-CM. **d** Comparison of the protein levels in HT-29 and Caco-2 cells. All data are presented as the mean±SD from three independent experiments. **P* <0.05.

**Supplementary figure 6 Blockage of MAPK/Erk signaling eliminated the proliferation of colon cancer cells induced by rhMMP1 a, b** The effect of Erk1/2 inhibitor (SCH772984) and an Akt inhibitor (MK-2206) on the growth and viability of HT-29 (**a**) and Caco-2 (**b**) cells treated with 20 μg/ml rhMMP1. **c** Western blotting analysis for the effect of SCH772984, MK-2206 and MK-5348 on cell cycle-related proteins and c-Myc and ETV4 in HT-29 and Caco-2 cells treated with 20 μg/ml rhMMP1. **d** Comparison of the protein levels in HT-29 and Caco-2 cells. All data are presented as the mean±SD from three independent experiments. **P* <0.05.
