# Supplementary material for: Tumor-associated macrophages (TAMs) depend on MMP1 for their cancer-promoting role
Source: Cell Death Discov. 2021 Nov 9;7:343. doi: 10.1038/s41420-021-00730-7 (PMC8578434; doi:10.1038/s41420-021-00730-7)
Supplement: Supplementary file 8 — Supplementary table [file 41420_2021_730_MOESM8_ESM.docx]

Supplementary Table 1 Primer sequence

| Gene | Sequence |
| --- | --- |
| RT-PCR | |
| cdc2 | F: 5’-TGATCCAGCCAAACGAATTTC-3’ |
|  | R: 5’-GCTACATCTTCTTAATCTGATTGTCCAA-3’ |
| cdc25a | F: 5’-GGCAAGCGTGTCATTGTTGTG-3’ |
|  | R: 5’-ACAGCTCAGGGTAGTGGAGTTTGG-3’ |
| CDK2 | F: 5’-GCTAGCAGACTTTGGACTAGCCAG-3’ |
|  | R: 5’-AGCTCGGTACCACAGGGTCA-3’ |
| CDK4 | F: 5’-ATGTTGTCCGGCTGATGGA-3’ |
|  | R: 5’-CACCAGGGTTACCTTGATCTCC-3’ |
| cylinA2 | F: 5’-GGATGGTAGTTTTGAGTCACCAC-3’ |
|  | R: 5’-CACGAGGATAGCTCTCATACTGT-3’ |
| cyclinB1 | F: 5’-TCTGGATAATGGTGAATGGACA-3’ |
|  | R: 5’-CGATGTGGCATACTTGTTCTTG-3’ |
| cyclinD1 | F: 5’-AAACAGATCATCCGCAAACAC-3’ |
|  | R: 5’-GTTGGGGCTCCTCAGGTTC-3’ |
| p16 | F: 5’-CATAGATGCCGCGGAAGGT-3’ |
|  | R: 5’-CCCGAGGTTTCTCAGAGCCT-3’ |
| p21 | F: 5’-GCAGACCAGCATGACAGATTTC-3’ |
|  | R: 5’-CGGATTAGGGCTTCCTCTTG-3’ |
| p53 | F: 5’-AAGAAACTGGCGGAATGGC-3’ |
|  | R: 5’-CCAAGAACCACCACCCCTGAGTC-3’ |
| GAPDH | F: 5’-TGCACCACCAACTGCTTAGC-3’ |
|  | R: 5’-GGCATGGACTGTGGTCATGAG-3’ |
| Luciferase Assays |  |
| F1(-1811 bp **-** +66 bp) | F: 5’-TCTCTGCCGCACCCTCCCTC-3’ |
| F2(-1199 bp **-** +66 bp) | F: 5’-TGGGCAGGAGATGCTAAATAAGATCTT-3’ |
| F3(-1038 bp **-** +66 bp) | F: 5’-TGGGCAAGGGGTGGGGAGTT-3’ |
| F4(-687 bp **-** +66 bp) | F: 5’-CCCTGCCTCGGCCTCCTGAA -3’ |
| F5(-412 bp **-** +66 bp) | F: 5’-AGCACTTTATGACCATCAGAACCAGTC-3’ |
| F6(-143 bp **-** +66 bp) | F: 5’-GCATGAGTCAGACAGCCTCTGG-3’ |
|  | R: 5’-CGCTGGGAAGCTGTGAGACACC-3’ |
| ChIP-qPCR  P1(-1811 bp **-** -1586 bp) | F: 5’-TCTCTGCCGCACCCTCCCTC-3’ |
|  | R: 5’-CCTGTTTTCTTTCTGCGTCAAGACTGA-3’ |
| P2(-1612 bp**-** -1512) | F: 5’-AGTCTTGACGCAGAAAGAAAACAGGA-3’ |
|  | R: 5’-CAGCACCTTATGGTGTCTCCCACC-3’ |
| P3(-1536 bp**-** -1426) | F: 5’-GGTGGGAGACACCATAAGGTGCTG -3’ |
|  | R: 5’-GCTCCCCTACTCATGCCCCACT-3’ |
| P4(-1448 bp**-** -1348) | F: 5’-AGTGGGGCATGAGTAGGGGAGC-3’ |
|  | R: 5’-TTGTCTTGGGTACTGGTGACCGGTGT-3’ |
| P5(-1374 bp**-** -1257 bp) | F: 5’-ACACCGGTCACCAGTACCCAAGACAA-3’ |
|  | F: 5’-AAGATCTTATTTAGCATCTCCTGCCCA-3’ |
| P6(-1284 bp**-** -1178 bp) | F: 5’-TGGGCAGGAGATGCTAAATAAGATCTT-3’ |
|  | F: 5’-CGCTCCTTGGTGCTAGGCAG-3’ |
| 3’-UTR | F: 5’-GGTGGGGGAGGGGAATCCAT-3’ |
|  | F: 5’-CTGCCCACCACCCAACTCTG-3’ |

Supplementary Table 2 Antibodies

| Antibodies | Source | | Identifier |
| --- | --- | --- | --- |
| cyclin A2 | | Abcam | Cat#ab181591 |
| cyclin B1 | | Abcam | Cat#ab32053 |
| cyclin D1 | | Abcam | Cat#ab40754 |
| cyclin E1 | | Abcam | Cat#ab33911 |
| p21^CIP1^ | | Abcam | Cat#ab109520 |
| FOXO1 | | Abcam | Cat#ab52857 |
| ETV4 | | Abcam | Cat#ab70425 |
| c-Myc | | Abcam | Cat#ab32072 |
| p-AKT^Ser473^ | | Abcam | Cat#ab81283 |
| AKT | | Abcam | Cat#ab8805 |
| Erk1/2 | | Cell Signaling Technology | Cat#4695 |
| p-Erk1/2^Thr202/Tyr204^ | | Cell Signaling Technology | Cat#8544 |
| Histone H3 | | Cell Signaling Technology | Cat#4499 |
